# RNase P-Associated External Guide Sequence Effectively Reduces the Expression of Human CC-Chemokine Receptor 5 and Inhibits the Infection of Human Immunodeficiency Virus 1

**DOI:** 10.1155/2013/509714

**Published:** 2012-12-27

**Authors:** Wenbo Zeng, Gia-Phong Vu, Yong Bai, Yuan-Chuan Chen, Phong Trang, Sangwei Lu, Gengfu Xiao, Fenyong Liu

**Affiliations:** ^1^State Key Laboratory of Virology, College of Life Sciences, Wuhan University, Hubei, Wuhan 430072, China; ^2^Program in Comparative Biochemistry, University of California, Berkeley, CA 94720, USA; ^3^School of Public Health, University of California, Berkeley, CA 94720, USA

## Abstract

External guide sequences (EGSs) represent a new class of RNA-based gene-targeting agents, consist of a sequence complementary to a target mRNA, and render the target RNA susceptible to degradation by ribonuclease P (RNase P). In this study, EGSs were constructed to target the mRNA encoding human CC-chemokine receptor 5 (CCR5), one of the primary coreceptors for HIV. An EGS RNA, C1, efficiently directed human RNase P to cleave the CCR5 mRNA sequence *in vitro*. A reduction of about 70% in the expression level of both CCR5 mRNA and protein and an inhibition of more than 50-fold in HIV (R5 strain Ba-L) p24 production were observed in cells that expressed C1. In comparison, a reduction of about 10% in the expression of CCR5 and viral growth was found in cells that either did not express the EGS or produced a “disabled” EGS which carried nucleotide mutations that precluded RNase P recognition. Furthermore, the same C1-expressing cells that were protected from R5 strain Ba-L retained susceptibility to X4 strain IIIB, which uses CXCR4 as the coreceptor instead of CCR5, suggesting that the RNase P-mediated cleavage induced by the EGS is specific for the target CCR5 but not the closely related CXCR4. Our results provide direct evidence that EGS RNAs against CCR5 are effective and specific in blocking HIV infection and growth. These results also demonstrate the feasibility to develop highly effective EGSs for anti-HIV therapy.

## 1. Introduction

Nucleic acid-based gene interference technologies are promising gene-targeting approaches for studying and treating human diseases [1–5]. For example, ribozymes have been shown to cleave viral mRNA sequences and inhibit viral replication in human cells [6–8]. More recently, small interfering RNAs (siRNAs) are effective in inducing endogenous RNase of the RNA-induced silencing complex (RISC) in the RNA interference (RNAi) pathway to inhibit gene expression and growth of several human viruses including HIV and human cytomegalovirus [1, 9–11]. Improving these current technologies and developing new nucleic acid-based strategies will provide important tools and reagents for basic research and clinical applications. 

**Figure 1:**
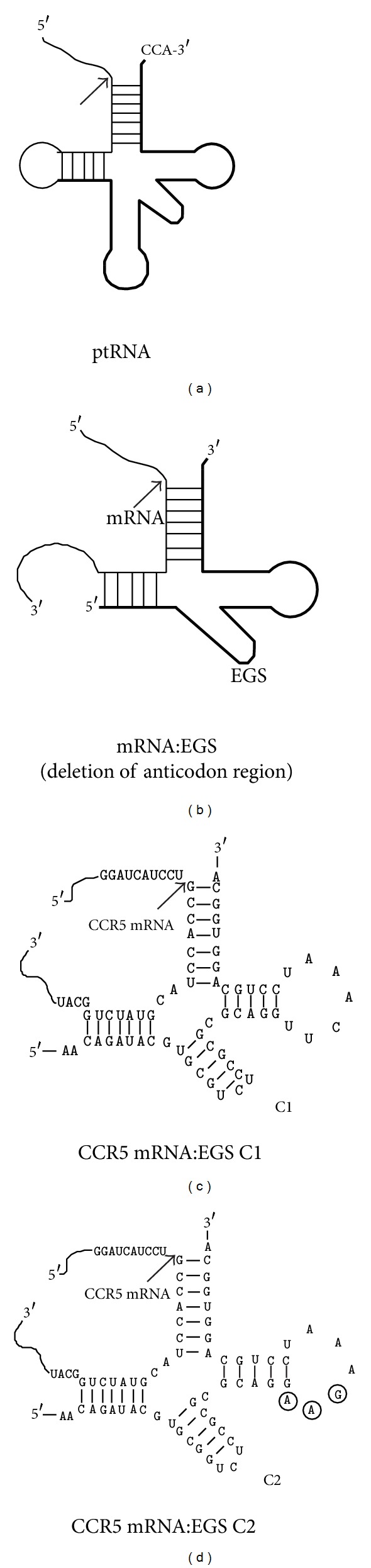
Schematic representation of substrates for RNase P. (a) A natural substrate (ptRNA). (b) A hybridized complex of a target RNA (e.g., mRNA) and a EGS that resembles the structure of a tRNA. (c) and (d) Complexes between CCR5 mRNA sequence and EGS C1 and C2, respectively. The sequences of C1 and C2 that were equivalent to the T-stem and loop and variable region of a tRNA molecule were derived from tRNA^ser^ [19]. Only the exact sequence of the CCR5 mRNA around the targeting site was shown. The EGS sequence is shown in bold. The site of cleavage by RNase P is marked with an arrowhead. The three nucleotides that are mutated in C2 are in circles.

Ribonuclease P (RNase P) has been proposed as a novel RNA-based gene interference strategy for downregulating gene expression [12, 13]. This enzyme is a ribonucleoprotein complex found in all organisms examined. It catalyzes a hydrolysis reaction to remove the leader sequence of precursor tRNA and is responsible for the maturation of 5′ termini of all tRNAs [14, 15]. RNase P recognizes the structures rather than the sequences of the substrates and therefore can hydrolyze different natural substrates. In a series of elegant studies, Altman and coworkers showed that a custom-designed external guide sequence (EGS) could bind to a target mRNA, forming a complex resembling a tRNA molecule, and directed RNase P for specific cleavage of the target RNA (Figures 1(a) and 1(b)) [16, 17]. The EGSs used to direct human RNase P for targeted cleavage consist of two sequence elements: a targeting sequence complementary to the mRNA sequence and a guide sequence, which is a portion of the natural tRNA sequence and is required for RNase P recognition (Figure 1(b)) [16, 17]. Subsequent studies have shown that expression of EGSs in human cells can reduce the expression of both cellular and viral genes [17–24]. For example, we have previously shown that EGSs efficiently direct human RNase P to cleave the mRNA sequence encoding the thymidine kinase (TK) of herpes simplex virus 1 (HSV-1) *in vitro* [19]. A reduction of about 70% in the TK mRNA and protein expression was observed in HSV-1 infected cells expressing the EGSs. 

Targeted cleavage of mRNA by RNase P using EGSs provides a unique approach to inactivate any RNA of known sequence expressed *in vivo*. Extensive studies exploiting the potential of ribozymes and RNAi molecules have been carried out, including several clinical trials using these technologies for treating certain human diseases [1, 4]. However, there are only a few studies evaluating the utility of the RNase P-based technology in targeting disease-associated mRNAs in human cells. In the present study, we constructed EGS RNAs to target the mRNA encoding human CC-chemokine receptor 5 (CCR5), which is one of the primary coreceptors for HIV [25, 26], and investigated their activity in blocking CCR5 expression and viral infection in HIV-infected cells. 

CCR5 belongs to the *β* subfamily of chemokine receptors and as such is not essential because of functional redundancies within this receptor family [27–30]. CCR5 and CXCR4 are two major coreceptors used by macrophage tropic (M-tropic or R5) and T-cell tropic (T-tropic or X4) HIV strains, respectively. In addition to CCR5 and CXCR4, other closely related chemokine receptors may also serve as coreceptors for some HIV-1 strains [27, 28]. Among these coreceptors, CCR5 appears to be often used by HIV-1 to establish initial infection. A naturally occurring 32 bp deletion in the CCR5 gene (CCR5Δ32) has a significant impact on both HIV-1 infection and progression to AIDS. Individuals who have a homozygous CCR5Δ32/CCR5Δ32 genotype (1%-2% of the Western European Caucasian population) appear to be relatively resistant to HIV infection [31–34]. In heterozygotes (CCR5Δ32/CCR5) (about 20% of the Western Caucasian population), the level of functional CCR5 is lower and disease progression to AIDS is usually slower than the homozygotes carrying the wild-type CCR5 gene (CCR5/CCR5) [35, 36]. These results suggest that elimination or even partial reduction of CCR5 may protect individuals from HIV-1 infection or slow down disease progression [5, 11]. Thus, CCR5 should represent an ideal target for anti-HIV gene therapy since CCR5 is not essential for normal physiological function and downregulation of CCR5 expression will impact the early stages of HIV-1 infection.

Recent studies showed that various nucleic acid-based gene interference approaches, including ribozymes and RNAi, against CCR5 are effective in blocking CCR5 expression and protecting cells from HIV infection [37–40]. These results indicate that downregulation of CCR5 expression using gene targeting approaches may represent a promising strategy for treatment and prevention of HIV infection. However, no studies on using EGSs to inhibit CCR5 expression for blocking HIV infection have been reported. In this study, we constructed EGSs to target the CCR5 mRNA and investigated their activities in downregulating CCR5 expression and blocking HIV infection. The target CCR5 sequence does not share sequence homology [27, 28] with other members of the CC-chemokine receptor family in order to avoid potential cross-targeting of other chemokine receptors by the anti-CCR5 EGSs. One of the constructed EGSs, C1, was active in directing RNase P to cleave the target mRNA *in vitro*. In cells expressed C1, a reduction of more than 70% in the CCR5 expression and an inhibition of at least 50-fold in infection of R5 HIV_Ba-L_ strain were observed. In contrast, a reduction of less than 10% in CCR5 expression and viral infection was observed in cells that either did not express an EGS or expressed an EGS that contained point mutations abolishing its ability to induce RNase P-mediated cleavage. Furthermore, the inhibition of HIV infection by EGS C1 appeared to be highly specific as the same cells that were protected from R5 strain Ba-L retained susceptibility to X4 strain IIIB, which uses CXCR4 as the coreceptor instead of CCR5. Our results provide the first direct evidence that EGS RNAs against CCR5 are highly effective and specific in blocking HIV infection and growth. These results also demonstrate the potential of generating highly active EGSs and using them as a research tool and as a therapeutic agent for gene-targeting applications.

## 2. Materials and Methods

### 2.1. Viruses and Cells

HIV R5 strain Ba-L (HIV_Ba-L_) and X4 strain IIIB (HIV_IIIB_) were kindly provided by Dr. Shibo Jiang (New York Blood Center). Human H9 and PM1 cells were obtained from the NIH AIDS Research and Reference Reagents Program. Viral stocks were titered on PM1 cells [41]. 

### 2.2. Construction of EGS RNAs and RNA Substrate for Studies *In Vitro*

The DNA sequence that encodes substrate ccr5-1 was constructed by PCR using pGEM3zf(+) as a template and oligonucleotides AF25 (5′-GGAATTCTAATACGACTCACTATAG-3′) and sCCR5 (5′-GAATACTATGCCAGATACGTAGGTGGCAGGATGATCCTATAGTGAGTCGTATTA-3′) as 5′ and 3′ primers, respectively. The DNA sequences coding for the EGSs were synthesized by the polymerase chain reaction (PCR), using construct pTK112 DNA [19] as the template. To construct the DNA that encodes EGS C1, the 5′ and 3′ primers were oligoC11 (5′-TAATACGACTCACTATAGGTTAACAGATACGTGCGGTCTCC-3′) and oligoC12 (5′-CCCGCTCGAGAAAAAATGCCACCUGCAGGATTTG-3′), respectively. To construct the DNA that encodes EGS C2, the 5′ and 3′ primers were oligoC11 and the oligoC21 (5′-CCCGCTCGAGAAAAAATGCCACCUGCAGGATTTCTTCCTGCGCGCG-3′), respectively. 

### 2.3. *In Vitro* Binding and Cleavage Reactions

Human RNase P was prepared from HeLa cellular extracts as described previously [17, 19, 21]. The EGSs and [^32^P]-labeled ccr5-1 were incubated with human RNase P at 37°C in buffer A (50 mM Tris, pH 7.4, 100 mM NH_4_Cl, and 10 mM MgCl_2_). Cleavage products were separated in denaturing gels and analyzed with an STORM840 phosphorimager. 

 The procedures to measure the equilibrium dissociation constants (*K*_*d*_) of complexes of the EGSs and the substrates were modified from Pyle et al. [42]. In brief, various concentrations of EGSs were preincubated in buffer B (50 mM Tris, pH 7.5, 100 mM NH_4_Cl, 10 mM MgCl_2_, 3% glycerol, 0.1% xylene cyanol, and 0.1% bromophenol blue) for 10 minutes before mixing with an equal volume of different concentrations of substrate RNA preheated under identical conditions. The samples were incubated for 10–120 minutes to allow binding, then loaded on a 5% polyacrylamide gel, and run at 10 Watts. The electrophoresis running buffer contained 100 mM Tris-Hepes pH 7.5 and 10 mM MgCl_2_ [42]. The value of *K*_*d*_ was then extrapolated from a graph plotting percent of product bound versus EGS concentration [21]. The values were the average of three experiments.

### 2.4. Construction of the EGS-Expressing Cell Lines

The DNA sequences coding for the EGSs were subcloned into retroviral vector LXSN and placed under the control of the U6 RNA promoter [43, 44]. The protocols to construct EGS-expressing cell lines were modified from Miller and Rosman [43]. In brief, the retroviral vector DNAs that contained the EGS sequence were transfected into amphotropic PA317 cells using a mammalian transfection kit (Invitrogen, San Diego, CA). Forty-eight hours after transfection, culture supernatants that contained retroviruses were collected and used to infect human PM1 cells. At 48–72 hours after infection, neomycin (Invitrogen) was added to the culture medium at a final concentration of 800 *μ*g/mL. Cells were subsequently selected in the presence of neomycin for three weeks, and neomycin-resistant cells were cloned. 

### 2.5. Studies of the Expression of EGS RNAs and CCR5

Northern analyses were used to determine the expression levels of EGS RNAs and CCR5 mRNA. The RNA fractions were separated in 0.8–2.5% agarose gels that contained formaldehyde, transferred to a nitrocellulose membrane, hybridized with the [^32^P]-radiolabeled DNA probes that contained the EGSs, CCR5, or human *β*-actin DNA sequences, and analyzed with a STORM840 phosphorimager. The radiolabeled DNA probes used to detect EGS RNAs, CCR5 mRNA, and human *β*-actin mRNA were synthesized using a random primed labeling kit (Roche Applied Science, Indianapolis, IN). The expression level of cell surface CCR5 was determined by fluorescence-activated cell sorter (FACS) analysis with a PE-conjugated anti-human CCR5 antibody, using an FACS VintageSE sorter (Bacton-Dickinson, San Jose, CA). The analysis procedures were carried out according to the manufacturer's recommendation. 

### 2.6. Studies of the Inhibition of Viral Infection

To study the EGS-mediated inhibition of viral infection, 5 × 10^5^ PM1 cells that either expressed EGSs or did not express an EGS were infected with HIV_Ba-L_ or HIV_IIIB_ at an MOI of 0.02–0.1 for a period of 3 hrs. The cells were washed and replated in RPMI medium that contains 10% fetal bovine serum, and were refed with one-half volume of fresh medium every 3 days. Culture media were harvested at 3-day intervals throughout 15 days after infection, and HIV production was monitored by assaying the level of p24 in culture supernatants with a p24 ELISA kit (Beckman-Coulter, Miami, FL). The values obtained were the average from triplicate experiments.

### 2.7. Determination of Levels of Total Intracellular HIV RNA

Total RNA samples were isolated from the cells at 48–72 hours after infection, using Trizol reagent (Invitrogen, San Diego, CA), treated with RQ1 DNase (Promega, Madison, WI), and then reverse-transcribed in the presence of PowerScript reverse transcriptase (Clontech, Palo Alto, CA). The resulting cDNA was added to PCR mix containing 1x titanium Taq PCR buffer, 1 mM dNTPs, SYBR Green (1 : 50,000), 10 nM fluorescein, 1x titanium Taq DNA polymerase (Clontech), and 20 pmol each of 5′ primer HIV-5 (5′-CATCCAGGAAGTCAGCCT-3′) and 3′ primer HIV-3 (5′-TTCCTGCCATAGGAGATGC-3′). Real-time PCR was carried out in an iCycler (Bio-Rad, Hercules, CA), and the PCR reaction consisted of 35 cycles with denaturation at 94°C for 40 seconds, followed by primer annealing at 50°C for 40 seconds and extension at 72°C for 40 seconds. To normalize the RNA level, the level of actin mRNA was assayed by real-time PCR using the same PCR mix except for the primers which were actin-5 (5′-TGACGGGGTCACCCACACTGTGCCCATCTA-3′) and actin-3 (5′-CTAGAAGCATTGCGGTGGCAGATGGAGGG-3′), respectively [45]. A standard (dilution) curve was generated by amplifying different dilutions of the RNA transcript of the *tat* sequence that was produced by an *in vitro* transcription kit with T7 RNA polymerase (Promega, Madison, WI). The real-time PCR results were derived from three independent experiments. 

## 3. Results

### 3.1. Design of EGSs and *In Vitro* Studies of Their Targeting Activity

Since most mRNA species inside cells are usually associated with proteins and are present in a highly organized and folded conformation, it is critical to choose a targeting region that is accessible to binding of EGSs in order to achieve efficient targeting. In previous studies, hairpin and hammerhead ribozymes were constructed to target the CCR5 mRNA, and the targeting sites were chosen based on the *in vitro* cleavage efficacy of the target mRNA by the constructed ribozymes in the absence of cellular proteins, combined wih computational analysis [37–40]. *In vivo* mapping with dimethyl sulphate (DMS) has been extensively used to determine the accessibility of mRNA and structure of RNAs in cells [44, 46]. Using this method, we mapped the region around the translation initiation site of CCR5 mRNA as this region is supposed to be accessible to ribosome binding [26]. A position, 29 nucleotides downstream from the CCR5 translational initiation codon, was chosen as the cleavage site for human RNase P. This site appeared to be one of the regions most accessible to DMS modification (data not shown). The target CCR5 sequence does not share sequence homology with other members of the CC-chemokine receptor family [27, 28], so potential cross-targeting of other chemokine receptors by the anti-CCR5 EGSs would be unlikely. Moreover, its flanking sequence exhibited several sequence features that need to be present in order to interact with an EGS and RNase P to achieve efficient cleavage. These features include that the nucleotides 3′ and 5′ adjacent to the site of cleavage are a guanosine and a pyrimidine, respectively [47]. The interactions of these sequence elements with the EGS facilitate the formation of the mRNA-EGS complex into a tRNA-like structure while those with RNase P are critical for recognition and cleavage by the enzyme. 

 Two EGSs were designed based on the sequence of tRNA^ser^ and constructed (Figures 1(c) and 1(d)) in a similar way as described previously [19, 47]. EGS C1 (Figure 1(c)) resembles a portion of tRNA^ser^ structure, which contains the variable region, the T stem and loop, and a part of the acceptor stem, with deletion of the anticodon region (Figure 1(b)). The anticodon domain has been shown to be not essential for EGS activity and actually inhibits EGS activity to target CAT and TK mRNAs for cleavage by human RNase P [19, 47]. The second EGS, C2, was derived from C1 by introducing point mutations (5′-UUC-3′ -> AAG) at the three highly conserved positions in the T-loop of C1 (Figure 1(d)). These nucleotides were found in most of the known natural tRNA sequences [48] and were believed to be important for the interactions between the tRNA domains and human RNase P [15]. Previous studies have shown that EGSs carrying these mutations precluded RNase P recognition and exhibited little activity in directing RNase P-mediated cleavage [21, 23, 47]. 

### 3.2. *In Vitro* Cleavage of the CCR5 mRNA Sequence by Human RNase P in the Presence of EGSs

**Figure 2:**
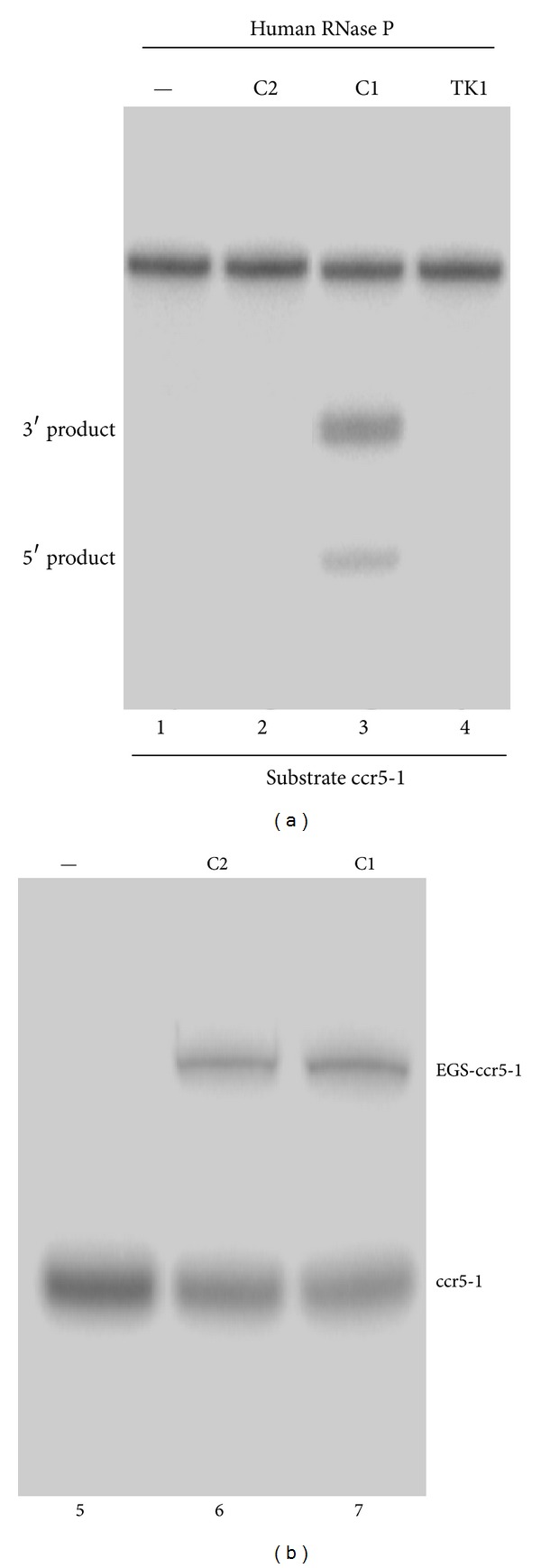
(a) Cleavage of CCR5 mRNA sequence (substrate ccr5-1) by human RNase P in the presence of different EGSs. No EGS was added to the reaction mixture in lane 1. 1 nM of the EGS C2 (lane 2), C1 (lane 3), and TK1 (lane 4) was incubated with [^32^P]-labeled CCR5 mRNA substrate (20 nM) and human RNase P (2 units) at 37°C in a volume of 10 *μ*L for 15 minutes in buffer A (50 mM Tris, pH 7.4, 100 mM NH_4_Cl, and 10 mM MgCl_2_). (b) *In vitro* binding of [^32^P]-labeled substrate ccr5-1 and EGSs. Substrate ccr5-1 at a concentration of 0.1 nM was incubated either alone (lane 5) or in the presence of 2 nM EGS C1 (lane 7) and C2 (lane 6) in buffer B for 15 min to allow binding and then loaded on a nondenaturing polyacrylamide gel. Experimental details can be found in Section 2.

The DNA sequences coding for the EGSs were generated by PCR using primers that contained the sequences complementary to the targeted region of the CCR5 mRNA and were under the control of the promoter for T7 RNA polymerase. EGS RNAs were synthesized* in vitro* from these DNA sequences by T7 RNA polymerase and subsequently incubated with human RNase P and substrate ccr5-1, which contains a 37-nucleotide long CCR5 mRNA sequence. In the absence of EGS RNAs (Figure 2, lane 1), no cleavage of CCR5 mRNA sequence was observed. Cleavage of substrate ccr5-1 by human RNase P was apparent in the presence of EGS C1 (Figure 2, lane 3). In contrast, cleavage of the same substrate was hardly detected in the presence of C2 (Figure 2, lane 2), consistent with the previous observations that the three highly conserved nucleotides in the T-loop that are mutated in C2 are important for recognition by RNase P and mutation of these nucleotides probably disrupts the interaction between RNase P and the EGSs [47, 49–51].

 Experiments were further carried out to determine whether the differential cleavage efficiencies observed with EGS C1 and C2 are possibly due to their different binding affinities to the CCR5 mRNA sequence. The binding between the EGS and substrate ccr5-1 was assayed in the absence of human RNase P, and the EGS-ccr5-1 complexes were separated in polyacrylamide gels under nondenaturing conditions. Similar amounts of complexes formed by these EGSs and the CCR5 mRNA sequence were observed when the same amount of EGSs was used (data not shown). Further detailed assays under different concentrations of the EGSs and the CCR5 mRNA sequence indicated that the binding affinity of C2 to substrate ccr5-1 (*K*_*d*_ = 800 ± 200 nM) is similar to that of C1 (*K*_*d*_ = 900 ± 150 nM). Meanwhile, very little amount of cleavage products was observed in the presence of C2 even under high concentrations of RNase P and prolonged incubation period (Figure 2, lane 2; data not shown). These observations indicated that the T-loop mutations do not significantly affect the binding affinity between C2 and CCR5 mRNA sequence but disrupt the recognition of EGS-CCR5 mRNA complex by RNase P. Thus, EGS C2 may be used as a control for the antisense effect in our experiments in cultured cells (see the following). 

An additional EGS, TK1, was also constructed and cloned into vector LXSN and under the control of the U6 RNA promoter. TK1 was designed to target the mRNA encoding the thymidine kinase (TK) of herpes simplex virus 1 (HSV-1), which has little sequence homology with the CCR5 gene or HIV-1 genome [19]. No RNase P-mediated cleavage of substrate ccr5-1 in the presence of TK1 was observed *in vitro* (Figure 2, lane 4). This EGS was used as the control to determine whether EGS RNA with an incorrect guide sequence could target the CCR5 mRNA sequence in tissue culture.

### 3.3. Expression of Anti-CCR5 EGSs in Human Cells

**Figure 3:**
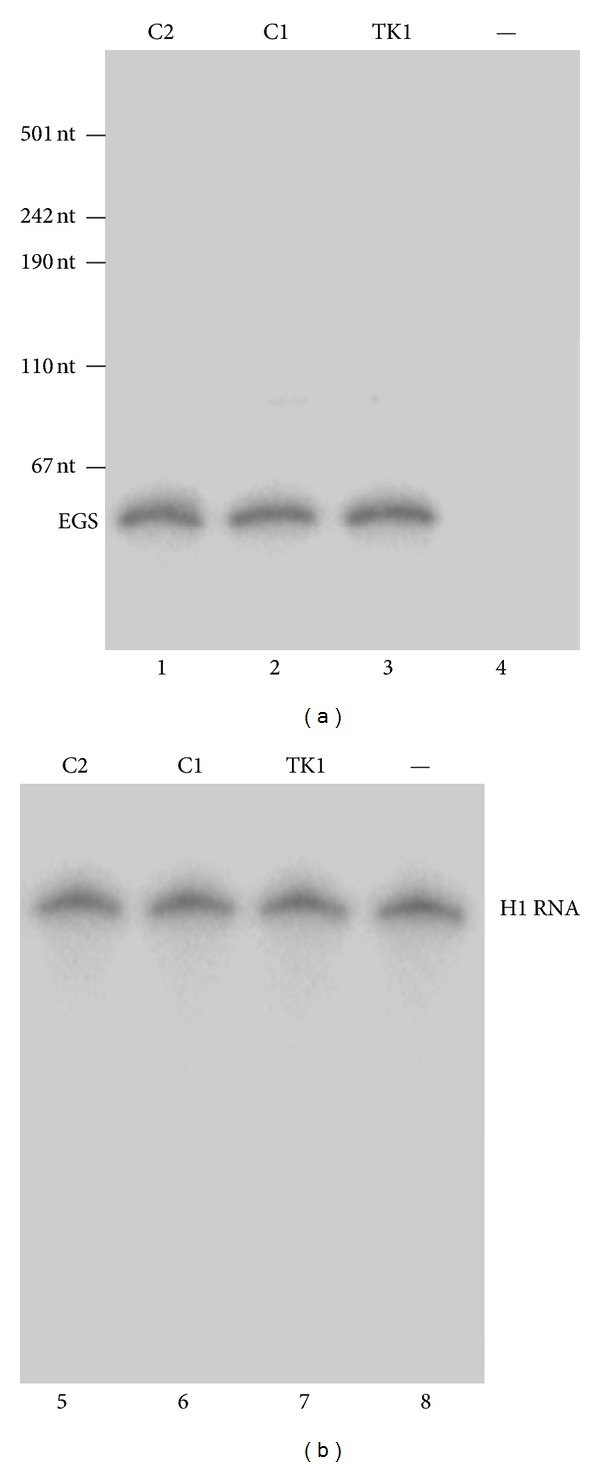
The expression of EGS RNAs in cultured cells. Northern analyses were carried out using nuclear RNA fractions isolated from parental PM1 cells (—, lanes 4 and 8) and a cloned cell line that expressed C2 (lanes 1 and 5), C1 (lanes 2 and 6), and TK1 (lanes 3 and 7). Equal amounts of each RNA sample (30 *μ*g) were separated on 2% agarose gels that contained formaldehyde, transferred to nitrocellulose membranes, and hybridized to a [^32^P]-radiolabeled probe that contained the DNA sequence coding for EGS C1 (lanes 1–4) or H1 RNA (lanes 5–8), the RNA subunit of human RNase P and a nuclear RNA [15]. The hybridized products corresponding to the full-length retroviral transcripts (~6 kb), transcribed from the LTR promoter, are at the top of the gel and are not shown.

The DNA sequences coding for the EGSs were subcloned into retroviral vector LXSN and placed under the control of the small nuclear U6 RNA promoter, which has previously been shown to express EGS RNA and other RNAs steadily [17, 44, 52]. To construct cell lines that express EGS RNAs, amphotropic packaging cells (PA317) [19, 43] were transfected with LXSN-C1, LXSN-C2, and LXSN-TK1 DNAs to produce retroviral vectors that contained the genes for the EGS RNAs. Human PM1 cells, which are permissive to HIV infection [41], were then infected with these vectors, and cells expressing the EGSs were cloned. The level of EGS RNA expression in each individual cell clone was determined by Northern analysis with DNA probes that were complementary to the EGSs, using the expression of human H1 RNA as the internal and loading control (Figure 3). Figure 3 shows the result from cloned cell lines that expressed EGS C1, C2, and TK1 (lanes 1–3 and 5–7). The expression level of human H1 RNA was similar in each lane in Figure 3(b), suggesting that an equal amount of cellular RNAs was present in each lane of the gel. The constructed lines and a control line in which cells were transfected with LXSN vector DNA alone were indistinguishable in terms of their growth and viability for up to two months (data not shown), suggesting that the expression of the EGSs did not exhibit significant cytotoxicity. Only the cell lines that expressed similar levels of these EGS RNAs were used for further studies in tissue culture. 

### 3.4. Inhibition of Human CCR5 Expression in EGS-Expressing Cells

**Figure 4:**
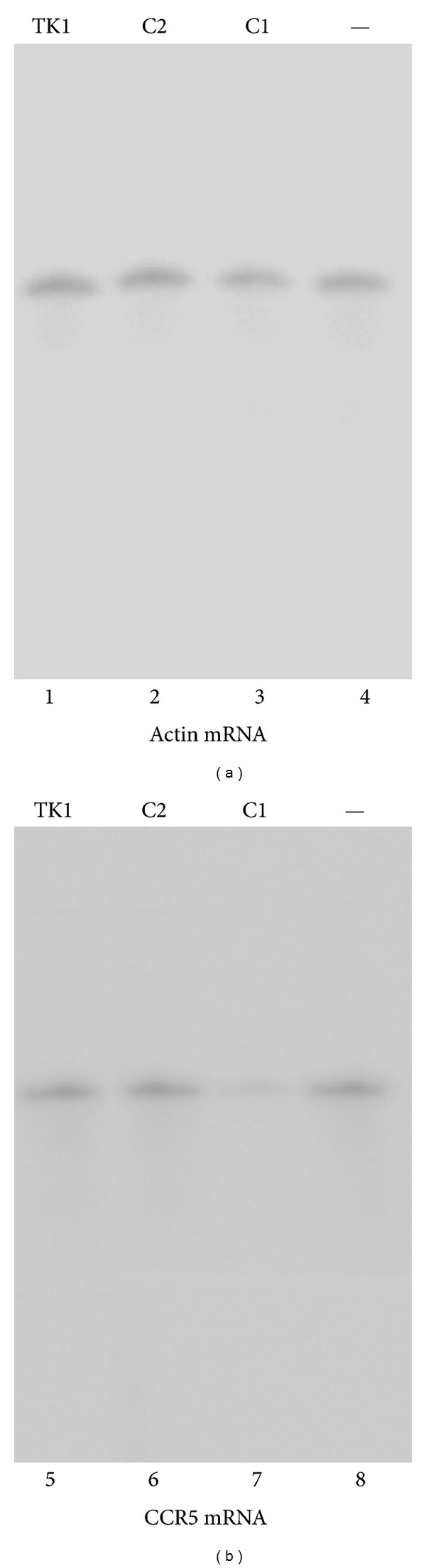
Levels of CCR5 mRNA. Northern analyses were carried out using RNA isolated from parental PM1 cells (—, lanes 4 and 8) and cell lines that expressed TK1 (lanes 1 and 5), C2 (lanes 2 and 6), and C1 (lanes 3 and 7). Equal amounts of each RNA sample (30 *μ*g) were separated on agarose gels that contained formaldehyde, transferred to nitrocellulose membranes, and hybridized to a [^32^P]-radiolabeled probe that contained the actin (lanes 1–4) and CCR5 mRNA sequences (lanes 5–8).

To determine if the EGSs inhibited the expression of CCR5, total RNAs were isolated from the EGS-expressing cells and the levels of CCR5 mRNA were determined by Northern analyses. The level of the mRNA encoding actin (actin mRNA) was used as an internal control for the quantitation of expression of CCR5 mRNA.  Figure 4 shows the results of the Northern analysis experiments with the CCR5 (Figure 4(b)) and actin RNA probes (Figure 4(a)). The expression level of human actin mRNA was similar in each lane in Figure 4(a), suggesting that an equal amount of cellular RNAs was present in each lane of the gel. A reduction of 70 ± 5% (average of three experiments) in the level of CCR5 mRNA expression was observed in cells that expressed EGS C1 (Figure 4, lane 7). In contrast, cells that expressed C2 only exhibited a reduction of 8 ± 4% (Figure 4, lane 6). No reduction in the expression level of CCR5 mRNA was observed in cells that expressed EGS TK1 (lane 5). The low level of inhibition found in cells that expressed C2 RNA was probably due to an antisense effect. This is because C2, with the point mutations at the T-loop (Figure 1(d)), exhibited little targeting activity but bound to the targeted CCR5 mRNA sequence as well as EGS C1. Thus, these observations suggest that the significant reduction of CCR5 mRNA expression in cells that expressed C1 was due to the RNase P-mediated cleavage of the target mRNA directed by the EGS. No products of the cleavage of CCR5 mRNA were detected in our Northern analyses presumably because these RNA products, which lacked either a cap structure or a polyA sequence, were rapidly degraded by intracellular RNases. 

**Figure 5:**
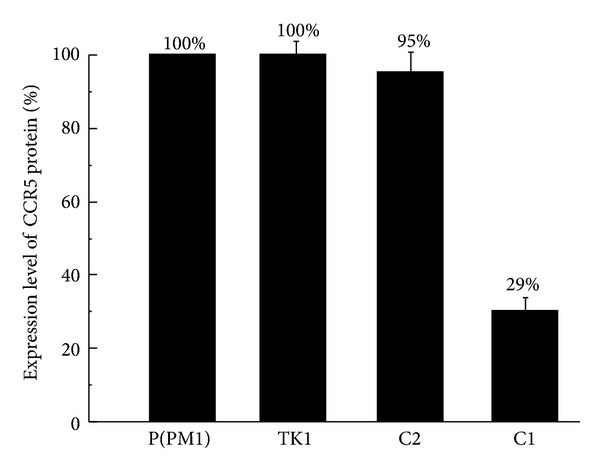
Fluorescence-activated cell sorting (FACS) analysis of CCR5 expression at the surface of the parental PM1 cells [P(PM1)] and cells that expressed EGS TK1 (TK1), C2 (C2), and C1 (C1). Quantitation was carried out by labeling the cells with a PE-conjugated anti-CCR5 antibody. The results are expressed in percent of fluorescence compared with the parental PM1 cells, which serve as the control. The values are the means from triplicate experiments. The standard deviation is indicated by the error bars.

We next measured the level of the cell surface CCR5 protein to determine whether the EGS-mediated reduction in the expression of CCR5 mRNA results in a decrease in CCR5 surface expression. The expression of surface CCR5 protein was quantified using fluorescence-activated cell sorter (FACS) analysis with a monoclonal antibody specifically against CCR5. A reduction of 71 ± 5% and 5 ± 3% in the surface expression of CCR5 was observed in cells that expressed C1 and C2, respectively, while no reduction in CCR5 expression was found in the TK1-expressing cells (Figure 5). Thus, the reduction in the surface expression of CCR5 protein correlates with the decrease in the intracellular expression of CCR5 mRNA. 

### 3.5. Inhibition of HIV Infection in the EGS-Expressing Cells

**Figure 6:**
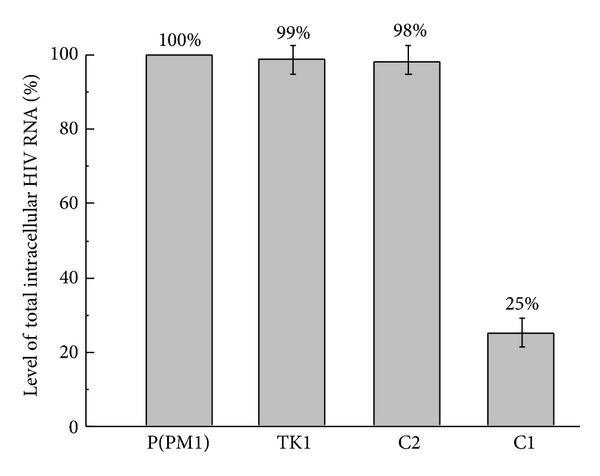
Schematic representation of the levels of total (unspliced and spliced) intracellular HIV RNA in HIV_Ba-L_-infected PM1 cells that did not express an EGS [P(PM1)] or expressed EGS C1 (C1), C2 (C2), and TK1 (TK1). The RNA samples were isolated from cells at 48 hours after infection, and the levels of HIV RNA were determined using a real-time PCR assay. The values shown are the averages from three independent experiments.

A reduction of CCR5 expression in the EGS-expressing cells is expected to result in a better protection of the cells from infection by an M-tropic HIV strain (e.g., HIV_Ba-L_), which uses CCR5 as the coreceptor for infection [29, 30]. To determine whether this was the case, cells were infected with HIV_Ba-L_ at a multiplicity of infection (MOI) of 0.02. RNA samples were isolated from cells at 48–72 hours postinfection. The levels of total HIV-1 intracellular (unspliced and spliced) RNA were determined with a real-time PCR assay, using the expression level of the actin mRNA as the internal control. The results of three independent experiments indicated that a reduction of 75% ± 7%, 2 ± 3%, and 1 ± 2% in the level of total HIV-1 intracellular RNA was observed in cells that expressed EGS C1, C2, and TK1, respectively (Figure 6). 

**Figure 7:**
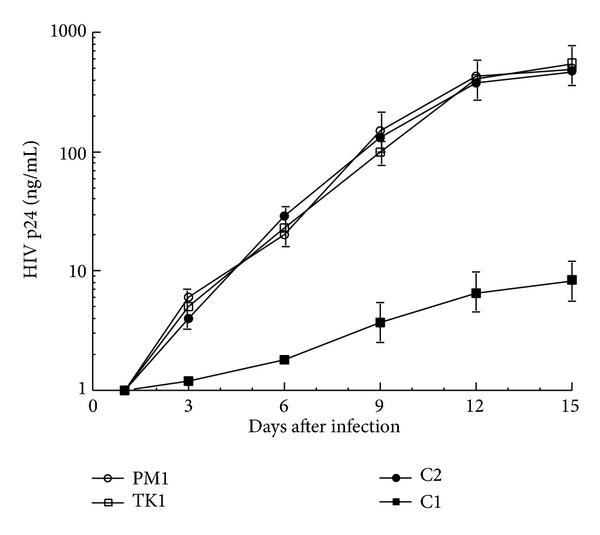
Growth of HIV-1 in PM1 cells and cell lines that expressed EGS RNAs. 5 × 10^5^ cells were infected with HIV-1 at a MOI of 0.02–0.1. Viral production was determined by a p24 antigen assay as a function of time postinfection. The values are the means from triplicate experiments. The standard deviation is indicated by the error bars.

The progress of the resultant infection was also followed by harvesting cultured media at different time points after infection and measuring the level of HIV p24 in the supernatants. The results, shown in Figure 7, indicated that EGS C1 but not C2 or TK1 was capable of substantially inhibiting HIV replication throughout the 15-day time course experiment. After 12 days after infection, a reduction of at least 50-fold in the level of HIV p24 was observed in cells that expressed EGS C1. In contrast, no significant reduction was found in those that expressed the control EGSs C2 or TK1 (Figure 7). Since EGS C2, with the point mutations at the T-loop (Figure 1(d)), exhibited little targeting activity but bound to the targeted CCR5 mRNA sequence as well as EGS C1, these observations suggest that the significant inhibition of HIV infection in cells that expressed C1 was due to the RNase P-mediated cleavage of the target CCR5 mRNA directed by the EGS. 

### 3.6. Specific Antiviral Activity of EGSs for M-Tropic but Not T-Tropic HIV Strain

**Figure 8:**
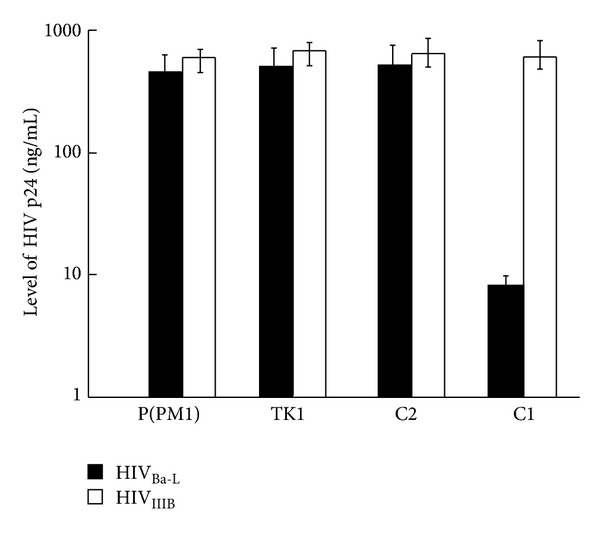
Schematic representation of the expression levels of supernatant HIV-1 p24 protein in viral infected cells that did not express an EGS [P(PM1)] or expressed EGS C1 (C1), C2 (C2), and TK1 (TK1). The cells were either infected with X4 strain HIV_Ba-L_ or R5 strain HIV_IIIB_. The protein samples were isolated from culture media at 15 days after infection. The levels of p24 protein were measured with an HIV p24 ELISA kit. The values shown are the averages from three independent experiments. Solid bars: HIV_Ba-L_; open bars: HIV_IIIB_.

Human PM1 cells, which were the parental cells used to produce EGS-expressing cell lines, expressed both CCR5 and CXCR4, which serve as the coreceptor for the M-tropic (e.g., HIV_Ba-L_) and T-tropic HIV strains (e.g., HIV_IIIB_), respectively [29, 41]. If a constructed EGS is specific in downregulating CCR5 expression and does not affect the expression of other cellular or viral proteins such as CXCR4, the cells that express the EGS should be only resistant to infection by M-tropic HIV strains and should remain susceptible to infection by T-tropic strains. To determine the specificity of the antiviral effect of the EGSs, EGS-expressing cells were infected with X4 strain HIV_IIIB_ and R5 strain HIV_Ba-L_. Viral infection was monitored by assaying the virion production on day 15 after infection with measuring the level of p24 in the culture supernatants (Figure 8). The C1-expressing cells were highly resistant to infection by HIV_Ba-L_. A reduction of about 75-fold in the level of p24 was observed in the C1-expressing cells that were infected with strain Ba-L (Figure 8). In contrast, these cells were still very susceptible to infection by HIV_IIIB_ as similar levels of HIV p24 were found in the strain IIIB-infected cells that either did not express an EGS or expressed C1, C2, and TK1 (Figure 8). Thus, the inhibition of viral replication by EGS C1 appeared to be highly specific as the same cells that were protected from R5 strain Ba-L retained susceptibility to X4 strain IIIB, which uses CXCR4 as the coreceptor instead of CCR5. Furthermore, our results suggest that the RNase P-mediated cleavage of the target mRNA directed by the EGS was required for the observed inhibition of HIV_Ba-L_ replication. This is because cells that expressed the control “disabled” EGS C2, which bound to the targeted CCR5 mRNA sequence as well as C1 but exhibited little targeting activity, were fully susceptible to infection by both HIV_Ba-L_ and HIV_IIIB_. 

## 4. Discussion

Nucleic acid-based gene interference strategies, such as antisense oligonucleotides, ribozymes or DNAzymes, and RNA interference (RNAi), represent powerful research tools and promising therapeutic agents for human diseases [1–5, 11]. Each of these approaches has its own advantages and limitations in term of targeting efficacy, sequence specificity, toxicity, and delivery efficiency *in vivo*. The EGS-based technology represents a unique approach for gene inactivation since it utilizes endogenous RNase P to generate highly efficient and specific cleavage of the target RNA [12, 13]. RNase P has been considered as one of the most ubiquitous and active enzymes found in nature as it is responsible for processing of all tRNA molecules, which accounts for 2% of total RNA species within a single cell [14, 15]. Moreover, RNase P-mediated cleavage directed by EGSs is specific and does not generate “irrelevant cleavage,” which is usually observed with RNase H-mediated cleavage induced by conventional antisense phosphothioate oligonucleotides [15, 53]. Thus, EGS molecules represent promising general gene-targeting agents that can be used in both basic research and clinical applications.

For the EGS-based technology to be successful as a therapeutic tool, the EGSs have to be highly active in targeting the mRNA for cleavage by RNase P and the mechanism of delivery of the EGSs has to be extremely efficient. We have constructed EGSs that target cellular CCR5 mRNA and shown that a functional EGS, C1, directed human RNase P to cleave the CCR5 mRNA sequence efficiently *in vitro. *Moreover, we showed that C1 reduced the expression levels of CCR5 mRNA and protein by about 70% and inhibited HIV infection by more than 50-fold in cells that expressed the EGS. In contrast, a reduction of less than 10% in the levels of CCR5 expression and HIV infection was observed in cells that expressed EGS C2 or TK1. TK1 targeted an unrelated mRNA and C2 bound efficiently to the CCR5 mRNA sequence but contained three-point mutations that disrupted RNase P recognition. Thus, the observed reduction in CCR5 expression and inhibition of HIV infection with EGS C1 is primarily attributed to the targeted cleavage by RNase P as opposed to the antisense effect or other nonspecific effects of the EGSs. 

Several lines of evidence presented in our study suggest that the targeting activity of the EGS may be specific. First, the presence of EGSs did not exhibit significant cytotoxicity as cells expressing EGSs are indistinguishable from the parental cells in terms of cell growth and viability for up to two months (data not shown). Second, the antiviral effect associated with the expression of EGS C1 RNA (inhibition of HIV infection) appears to be due to the reduction of CCR5 expression. This is because the expression of cellular surface CCR5 was found to be significantly reduced in cells that expressed EGS C1 but not in those that expressed EGS C2 and TK1 (Figures 4 and 5). The extent of the observed inhibition of the expression of cellular surface CCR5 protein correlates with that of the inhibition of the expression of intracellular CCR5 mRNA. Third, expression of EGS C1 only inhibits the expression of CCR5. No reductions in the expression levels of other cellular genes examined such as human H1 RNA, actin, and CXCR4 were observed in C1-expressing cells (Figure 4, data not shown). Cells expressing EGS C1 appeared to be resistant to infection by M-tropic HIV_Ba-L_ strain but remain very susceptible to infection by T tropic HIV_IIIB_ strain, suggesting that C1 specifically targeted CCR5 but not CXCR4. Thus, the EGS is specific in inhibiting the expression of its target mRNA.

To successfully use the RNase P-based technology for clinical applications, the EGSs need to be delivered specifically into target tissues and cell types. In our study, using a retroviral expression vector, the EGSs were delivered into cultured cells and were stably expressed. The promoter for small nuclear U6 RNA, which has been extensively used to express functional RNAs for gene targeting applications [17–19, 39, 52], was used for the expression of these EGSs. The efficient delivery and proper localization of the EGS in cells may be mediated by cellular tRNA-binding proteins, which may interact with the tRNA-like domains of the EGS and target the EGS for interactions with RNase P, a tRNA-processing enzyme [15]. Further studies on developing novel viral expression vectors, including lentiviral and adeno-associated virus-based expression vectors, as well as using tissue and cell-type-specific promoter expression cassettes will lead to specific and efficient delivery and expression of the EGSs in a target tissue and cell type. 

HIV, the etiological agent for AIDS [54, 55], primarily infects T cells and macrophages by using CXCR4 and CCR5 as the primary coreceptors, respectively [26, 30]. In order to prevent infection in these cells, one of the promising approaches for gene therapy against HIV infection is to construct EGSs against CCR5 and CXCR4 and to deliver and express these EGSs together in hematopoietic stem/progenitor cells before they differentiate into multilineage progeny cells *in vivo*, including T cells and macrophages [4, 5, 11, 56, 57]. One potential problem associated with anti-HIV therapy is the emergence of resistant mutants, which may ultimately render the currently available highly active antiretroviral therapy (HAART) ineffective [58–60]. The anti-CCR5 EGS-based gene therapy approach described in our study is aimed at a cellular gene, which does not have the mutagenic potential of the HIV genome. Moreover, the anti-CCR5 EGS strategy targets at the entry step of HIV prior to the step of genome integration and the start of the replication [54, 55], therefore should limit the possibility of the generation of the viral drug resistant mutations. 

Previous studies showed that various nucleic acid-based gene interference approaches, including ribozymes and RNAi, against CCR5 are effective in blocking CCR5 expression and protecting cells from HIV infection [37–40]. These results indicate that downregulation of CCR5 expression using gene targeting approaches may represent a promising strategy for treatment and prevention of HIV infection. We showed a reduction of about 70% in CCR5 expression and a reduction of more than 50-fold in p24 expression. The levels of inhibition of CCR5 expression observed in our study are similar to those observed in previous studies using hairpin and hammerhead ribozymes against CCR5 mRNA [37–40]. We note that an inhibition of less than 100-fold in p24 expression, as observed in our study, may not be sufficient to block HIV infection and therefore may not be significant in HIV therapy. EGS variants that were more efficient in inducing RNase P to cleave a target mRNA than tRNA-derived EGSs were generated by *in vitro* selection procedures and were shown to be more effective in blocking the expression of their target mRNAs in cultured cells [21, 47]. Further studies on *in vitro* genetic engineering and different designs of EGSs for increasing their targeting activity [21, 47] are needed in order to increase the efficacy of the EGSs *in vivo*. Recent studies have suggested that a combination of different therapeutic agents, such as a combination of ribozymes and RNAi, may offer one of the most promising approaches for gene therapy against HIV infection [5, 11, 57]. Thus, EGSs can be used in combination with currently available therapeutic strategies for maximal inhibition of HIV infection. These studies will further facilitate the development of the EGS-based technology for gene-targeting applications in both basic research and clinical application, including gene therapy for HIV infection.

## References

[1] Scherer LJ, Rossi JJ (2003). Approaches for the sequence-specific knockdown of mRNA. *Nature Biotechnology*.

[2] Santoro SW, Joyce GF (1997). A general purpose RNA-cleaving DNA enzyme. *Proceedings of the National Academy of Sciences of the United States of America*.

[3] Stein CA, Cheng YC (1993). Antisense oligonucleotides as therapeutic agents—is the bullet really magical?. *Science*.

[4] Wong-Staal F, Poeschla EM, Looney DJ (1998). A controlled, Phase 1 clinical trial to evaluate the safety and effects in HIV-1 infected humans of autologous lymphocytes transduced with a ribozyme that cleaves HIV-1 RNA. *Human Gene Therapy*.

[5] Zhou J, Rossi JJ (2011). Current progress in the development of RNAi-based therapeutics for HIV-1. *Gene Therapy*.

[6] Zu Putlitz J, Yu Q, Burke JM, Wands JR (1999). Combinatorial screening and intracellular antiviral activity of hairpin ribozymes directed against hepatitis B virus. *Journal of Virology*.

[7] Yu M, Ojwang J, Yamada O (1993). A hairpin ribozyme inhibits expression of diverse strains of human immunodeficiency virus type 1. *Proceedings of the National Academy of Sciences of the United States of America*.

[8] Sarver N, Cantin EM, Chang PS (1990). Ribozymes as potential anti-HIV-1 therapeutic agents. *Science*.

[9] Wiebusch L, Truss M, Hagemeier C (2004). Inhibition of human cytomegalovirus replication by small interfering RNAs. *Journal of General Virology*.

[10] Jacque JM, Triques K, Stevenson M (2002). Modulation of HIV-1 replication by RNA interference. *Nature*.

[11] Chung J, Rossi JJ, Jung U (2011). Current progress and challenges in HIV gene therapy. *Future Virology*.

[12] Gopalan V, Vioque A, Altman S (2002). RNase P: variations and uses. *Journal of Biological Chemistry*.

[13] Raj SML, Liu F (2003). Engineering of RNase P ribozyme for gene-targeting applications. *Gene*.

[14] Evans D, Marquez SM, Pace NR (2006). RNase P: interface of the RNA and protein worlds. *Trends in Biochemical Sciences*.

[15] Gopalan V, Altman S, Gesteland R, Cech T, Atkins J (2006). RNase P: structure and catalysis. *The RNA World*.

[16] Forster AC, Altman S (1990). External guide sequence for an RNA enzyme. *Science*.

[17] Yuan Y, Hwang ES, Altman S (1992). Targeted cleavage of mRNA by human RNase P. *Proceedings of the National Academy of Sciences of the United States of America*.

[18] Plehn-Dujowich D, Altman S (1998). Effective inhibition of influenza virus production in cultured cells by external guide sequences and ribonuclease P. *Proceedings of the National Academy of Sciences of the United States of America*.

[19] Kawa D, Wang J, Yuan Y, Liu F (1998). Inhibition of viral gene expression by human ribonuclease P. *RNA*.

[20] Guerrier-Takada C, Li Y, Altman S (1995). Artificial regulation of gene expression in *Escherichia coli* by RNase P. *Proceedings of the National Academy of Sciences of the United States of America*.

[21] Zhou T, Kim J, Kilani AF (2002). In vitro selection of external guide sequences for directing RNase P-mediated inhibition of viral gene expression. *Journal of Biological Chemistry*.

[22] Li H, Trang P, Kim K, Zhou T, Umamoto S, Liu F (2006). Effective inhibition of human cytomegalovirus gene expression and growth by intracellular expression of external guide sequence RNA. *RNA*.

[23] Zhu J, Trang P, Kim K, Zhou T, Deng H, Liu F (2004). Effective inhibition of Rta expression and lytic replication of Kaposi’s sarcoma-associated herpesvirus by human RNase P. *Proceedings of the National Academy of Sciences of the United States of America*.

[24] Kraus G, Geffin R, Spruill G (2002). Cross-clade inhibition of HIV-1 replication and cytopathology by using RNase P-associated external guide sequences. *Proceedings of the National Academy of Sciences of the United States of America*.

[25] Doranz BJ, Berson JF, Rucker J, Doms RW (1997). Chemokine receptors as fusion cofactors fat human immunodeficiency virus type 1 (HIV-1). *Immunologic Research*.

[26] Berger EA (1997). HIV entry and tropism: the chemokine receptor connection. *AIDS*.

[27] Deng HK, Unutmaz D, Kewalramani VN, Littman DR (1997). Expression cloning of new receptors used by simian and human immunodeficiency viruses. *Nature*.

[28] Liao F, Lee HH, Farber JM (1997). Cloning of STRL22, a new human gene encoding a G-protein-coupled receptor related to chemokine receptors and located on chromosome 6q27. *Genomics*.

[29] Berger EA, Murphy PM, Farber JM (1999). Chemokine receptors as HIV-1 coreceptors: roles in viral entry, tropism, and disease. *Annual Review of Immunology*.

[30] Bieniasz PD, Cullen BR (1998). Chemokine receptors and human immunodeficiency virus infection. *Frontiers in Bioscience*.

[31] Paxton WA, Martin SR, Tse D (1996). Relative resistance to HIV-1 infection of CD4 lymphocytes from persons who remain uninfected despite multiple high-risk sexual exposures. *Nature Medicine*.

[32] Samson M, Libert F, Doranz BJ (1996). Resistance to HIV-1 infection in caucasian individuals bearing mutant alleles of the CCR-5 chemokine receptor gene. *Nature*.

[33] Liu R, Paxton WA, Choe S (1996). Homozygous defect in HIV-1 coreceptor accounts for resistance of some multiply-exposed individuals to HIV-1 infection. *Cell*.

[34] Michael NL, Louie LG, Sheppard HW (1997). CCR5-delta 32 gene deletion in HIV-1 infected patients. *The Lancet*.

[35] Dean M, Carrington M, Winkler C (1996). Genetic restriction of HIV-1 infection and progression to AIDS by a deletion allele of the CKR5 structural gene. Hemophilia Growth and Development Study, Multicenter AIDS Cohort Study, Multicenter Hemophilia Cohort Study, San Francisco City Cohort, ALIVE Study. *Science*.

[36] Buseyne F, Janvier G, Teglas JP (1998). Impact of heterozygosity for the chemokine receptor CCR5 32-bp-deleted allele on plasma virus load and CD4 T lymphocytes in perinatally human immunodeficiency virus-infected children at 8 years of age. *Journal of Infectious Diseases*.

[37] Feng Y, Leavitt M, Tritz R (2000). Inhibition of CCR5-dependent HIV-1 infection by hairpin ribozyme gene therapy against CC-chemokine receptor 5. *Virology*.

[38] Cagnon L, Rossi JJ (2000). Downregulation of the CCR5 *β*-chemokine receptor and inhibition of HIV-1 infection by stable VA1-ribozyme chimeric transcripts. *Antisense and Nucleic Acid Drug Development*.

[39] Anderson J, Akkina R (2005). CXCR4 and CCR5 shRNA transgenic CD34+ cell derived macrophages are functionally normal and resist HIV-I infection. *Retrovirology*.

[40] Song E, Lee SK, Dykxhoorn DM (2003). Sustained small interfering RNA-mediated human immunodeficiency virus type 1 inhibition in primary macrophages. *Journal of Virology*.

[41] Lusso P, Cocchi F, Balotta C (1995). Growth of macrophage-tropic and primary human immunodeficiency virus type 1 (HIV-1) isolates in a unique CD4^+^ T-cell clone (PM1): failure to downregulate CD4 and to interfere with cell-line-tropic HIV-1. *Journal of Virology*.

[42] Pyle AM, McSwiggen JA, Cech TR (1990). Direct measurement of oligonucleotide substrate binding to wild-type and mutant ribozymes from Tetrahymena. *Proceedings of the National Academy of Sciences of the United States of America*.

[43] Miller AD, Rosman GJ (1989). Improved retroviral vectors for gene transfer and expression. *BioTechniques*.

[44] Liu F, Altman S (1995). Inhibition of viral gene expression by the catalytic RNA subunit of RNase P from *Escherichia coli*. *Genes and Development*.

[45] Daftarian PM, Kumar A, Kryworuchko M, Diaz-Mitoma F (1996). IL-10 production is enhanced in human T cells by IL-12 and IL-6 and in monocytes by tumor necrosis factor-*α*. *Journal of Immunology*.

[46] Zaug AJ, Cech TR (1995). Analysis of the structure of Tetrahymena nuclear RNAs in vivo: telomerase RNA, the self-splicing rRNA intron, and U2 snRNA. *RNA*.

[47] Yuan Y, Altman S (1994). Selection of guide sequences that direct efficient cleavage of mRNA by human ribonuclease P. *Science*.

[48] Sprinzl M, Dank N, Nock S, Schon A (1991). Compilation of tRNA sequences and sequences of tRNA genes. *Nucleic Acids Research*.

[49] Nolan JM, Burke DH, Pace NR (1993). Circularly permuted tRNAs as specific photoaffinity probes of ribonuclease P RNA structure. *Science*.

[50] Kahle D, Wehmeyer U, Krupp G (1990). Substrate recognition by RNase P and by the catalytic M1 RNA: identification of possible contact points in pre-tRNAs. *EMBO Journal*.

[51] Liu F, Altman S (1994). Differential evolution of substrates for an RNA enzyme in the presence and absence of its protein cofactor. *Cell*.

[52] Bertrand E, Castanotto D, Zhou C (1997). The expression cassette determines the functional activity of ribozymes in mammalian cells by controlling their intracellular localization. *RNA*.

[53] Ma M, Benimetskaya L, Lebedeva I, Dignam J, Takle G, Stein CA (2000). Intracellular mRNA cleavage induced through activation of RNase P by nuclease-resistant external guide sequences. *Nature Biotechnology*.

[54] Freed EO, Martin MA, Knipe DM, Howley PM, Griffin DE (2007). HIVs and their replication. *Fields Virology*.

[55] Kuritzkes DR, Walker BD, Knipe DM, Howley PM, Griffin DE (2007). HIV-1: pathogenesis, clinical manifestations, and treatment. *Fields Virology*.

[56] Akkina R, Banerjea A, Bai J, Anderson J, Li MJ, Rossi J (2003). siRNAs, ribozymes and RNA decoys in modeling stem cell-based gene therapy for HIV/AIDS. *Anticancer Research*.

[57] Strayer DS, Akkina R, Bunnell BA (2005). Current status of gene therapy strategies to treat HIV/AIDS. *Molecular Therapy*.

[58] Autran B, Carcelain G, Li TS (1997). Positive effects of combined antiretroviral therapy on CD4^+^ T cell homeostasis and function in advanced HIV disease. *Science*.

[59] Yeni P (2006). Update on HAART in HIV. *Journal of Hepatology*.

[60] Palella FJ, Delaney KM, Moorman AC (1998). Declining morbidity and mortality among patients with advanced human immunodeficiency virus infection. *New England Journal of Medicine*.

